# Free versus pedicled flaps for reconstruction of head and neck cancer defects: a systematic review

**DOI:** 10.1186/s40463-019-0334-y

**Published:** 2019-03-14

**Authors:** Fanny Gabrysz-Forget, Paul Tabet, Akram Rahal, Eric Bissada, Apostolos Christopoulos, Tareck Ayad

**Affiliations:** 1 Faculty of Medicine, Université de Montreal, Montreal, QC Canada; 2 Division of Otolaryngology - Head & Neck Surgery service, Université de Montréal, Montreal, QC Canada; 3 Department of Surgery, Centre Hospitalier de l’Université de Montréal, 900, Saint-Denis St. pavillon R, H2X 0A9, Montreal, Canada

**Keywords:** Flaps, Oncology, Reconstruction, Surgery, Outcomes

## Abstract

**Objective:**

The present review focuses on comparative studies of reconstruction with free flaps (FF) versus pedicled flaps (PF) after oncologic resection.

**Method:**

A systematic review was developed in compliance with PRISMA guidelines and performed using the Pubmed, Medline, EMBASE, Amed and Biosis databases.

**Results:**

A total of 30 articles were included. FF are associated with a longer operative time, a higher cost and a higher incidence of postoperative revisions compared to PF. FF are associated with a longer stay at the intensive care unit than the supraclavicular artery island flap (SCAIF) and with a more extended hospital stay compared to the submental island flap (SMIF). FF are associated with fewer infections and necrosis compared to the pectoralis major myocutaneous flap (PMMF).

**Conclusion:**

The comparison of both type of flaps is limited by the inherent design of the studies included. In sum, FF seem superior to the PMMF for several outcomes. SMIF and SCAIF compare favorably to FF for some specific indications achieving similar outcomes at a lower cost.

## Introduction

Head and neck reconstruction surgery has considerably evolved over the past decades, along with the trend of using either a free or a pedicled flap for the reconstruction of oncologic defects. Tracing back the history of flaps, the first pedicled flap (PF) was described by Susruta in 800 BC and consisted of a forehead flap [1]. It was later popularized by McGregor in 1963 and marked a turning point in reconstructive surgery, being the first ever reliable transposition flap [2]. A decade later, the pectoralis major myocutaneous flap (PMMF), supplied by the pectoral branch of the thoracoacromial artery, was introduced by Ariyan in 1979 [3]. The PMMF became the flap of choice for head and neck reconstruction in many centers and was extensively studied. However, concerns regarding the reliability of this flap for some defects resulted in the emergence of free flaps and other regional pedicled flaps, such as the supraclavicular artery island flap (SCAIF) and the submental island flap (SMIF).

With the advent of microvascular surgery in the 1970s, harvesting free flaps became popular in head and neck reconstruction surgery. Free tissue transfer was described by various authors, such as Daniel and Taylor who described the first cutaneous free flap in 1973 [4]. Free flap (FF) reconstruction slowly gained popularity over time to become the standard of care for large head & neck defects.

Free flaps require the expertise of microvascular surgery and longer operative times, but they show more versatility and robustness than PF for some defects [5, 6]. Pedicled flaps are accessible to both academic and community surgeons and considered more reliable in specific settings but are not suitable for every defect [7, 8].

Flap selection is a complex process, with FF and PF having both their respective pros and cons. More importantly, patients pre-operative conditions, the nature of the disease, and the available resources are significant factors to consider when choosing the appropriate reconstructive technique.

Favoring one type over the other to obtain the best outcomes is a challenge and a source of debate in the literature. The purpose of this study is to review all articles explicitly comparing FF to PF for head and neck defects reconstruction regarding demographic parameters, risk factors, tumor staging, operative time, hospitalization length, cost, post-operative complications, and outcomes, in order to better characterize the benefits and disadvantages of these flaps. Regarding post-operative complications, donor and recipient sites morbidity, as well as the impact of either FF or PF reconstruction techniques on patients’ quality of life, was evaluated to facilitate the choice for clinicians in the future.

## Materials and methods

### Literature review

The systematic review was performed in accordance with PRISMA guidelines, and a formal PROSPERO protocol was published according to the NHS International Prospective Register of Systematic Review (PROSPERO #42017055252). The Pubmed, Ovid-MEDLINE, EMBASE, Amed and Biosis databases were used to perform a literature review of English-language publication dating from 1948 to February 2017. Keyword combination included: free flaps AND pedicled flaps AND head and neck AND reconstruction surgery. The comparative study option was used as a limit to refine the search. Additionally, references in all articles were manually searched to identify other articles.

### Selection criteria

Prospective and retrospective articles explicitly comparing the use of free flap versus pedicled flaps for head and neck oncologic defects were included. The data compared had to include one of the following parameters: demographic characteristics, risk factors, radiation or chemotherapy use, operative time, length of stay, total cost, post-operative complications and outcomes concerning survival and quality of life. The paediatric population was excluded. Articles describing only revision surgery were also excluded.

**Fig. 1:**
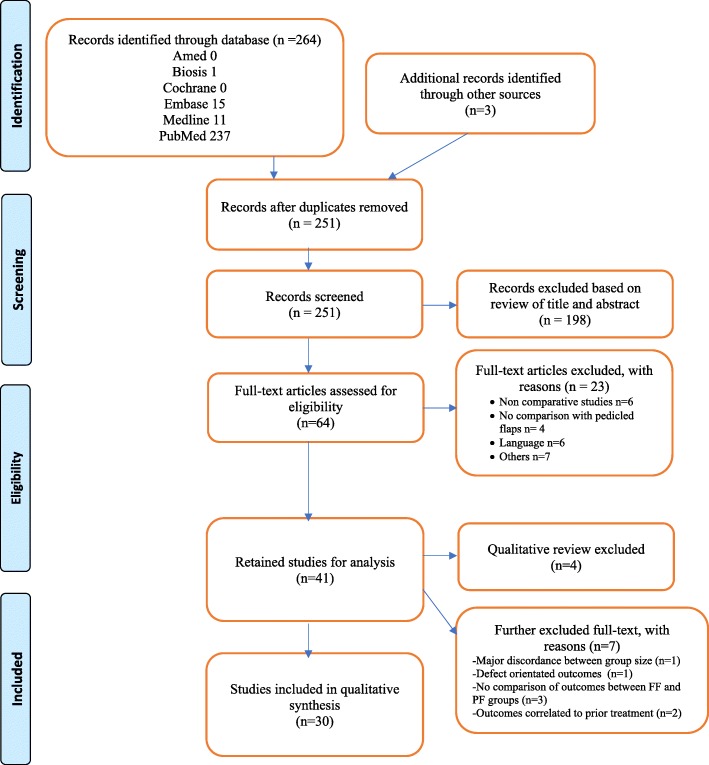
PRISMA flow diagram presenting the systematic review process

Titles and abstracts were initially screened by two investigators (F.G.F and P.T) to discard irrelevant studies. All reference lists of identified studies were then further analyzed to include any additional articles of interest. (Fig. 1). Selection of relevant studies was determined independently based on inclusion criteria. Any disagreement between reviewers was solved by discussions among the authors to reach consensus or by a third party (T.A), if necessary. The selection process was conducted per PRISMA guidelines. (Fig. 1).

### Quality assessment

The methodological quality of evidence and the risks of bias of the included studies were assessed with the MINORS criteria (Methodological Index for Non-randomized Studies) [9]. Twelve criteria are used to evaluate the level of evidence of comparative studies. Criteria are graded from 0 to 2 (0: not reported; 1: reported but inadequate; 2: reported and adequate), for a global ideal score of 24. Studies with MINORS score > 18 were considered to have low risk bias. Quality assessment was conducted independently by two investigators (F.G.F and P.T) and discrepancies were resolved through a mutual re-review.

## Results

A total of 30 articles were included for qualitative analysis after selection process (Fig. 1). All studies were retrospective except for one.

### Types of flaps

Of the included studies, 53.3% (*n* = 16) compared FF to PMMF. Ten percent compared FF to supraclavicular artery island flap (SCAIF) (*n* = 3) and 10% compared FF to submental artery island flap (SMIF) (*n* = 3). The other studies compared FF to an array of different pedicled flaps or unspecified pedicled flaps. Types of flaps of all included studies are detailed in Table 1.

**Table 1:** Overview of the included studies

Article	Ref	Study type	MINORS Score^a^	N total	n FF	n PF	Type of free flaps	Type of pedicled flaps	Defect / tumor location
Sinha, 2017 [14]	A	Retrospective review	20	517	384	133	ALT, FibF, RFFF	PMMF, SCAIF, SMIF	N/A
Goyal, 2017 [10]	B	Retrospective review	18	797	589	208	NA	PMMF, SCAIF, SMIF, TEMP, TRPF, DELT	Cutaneous/ skull base
									Oral cavity
									Oropharynx
									Larynx / hypopharynx
									Mandibular
									Sinonasal
									Composite/multiple sites
Li, 2016 [15]	C	Retrospective review	19	41	24	17	RFFF	PMMF	Oral cavity
Kozin, 2016 [5]	D	Retrospective review	20	72	28	45	RFFF, ALT	SCAIF	Cutaneous defect
									Parotid/temporal bone
Howard, 2016 [21]	E	Retrospective review	18	31	9	16	ALT	SMIF	Lateral skull base
						6		PLDF	
Geiger, 2016 [17]	F	Retrospective review	20	105	50	55	39 RFFF, 3 ALT, 4 FibF, 2 theLDF, 2 SFF, 2 ORFF	51 PMMF, 2 DIEP, 2 TPF	N/A
Gao, 2016 [49]	G	Retrospective review	16	60	34	26	RFFF	PMMF	N/A
Forner, 2016 [27]	H	Retrospective review	20	21	12	9	RFFF	SMIF	Oral cavity
									Oropharynx
Zhang S, 2015 [32]	I	Retrospective review	19	37	15	12	RFFF	SCAIF	Oral cavity
Zhang X, 2014 [16]	J	Retrospective review	19	110	79	31	ALT	PMMF	Oral cavity
									Oropharynx
Jing, 2014 [22]	K	Retrospective review	17	49	22	27	GFMF	PMMF	Larynx
Granzow, 2013 [7]	L	Retrospective review	18	34	16	18	FFF	SCAIF	Larynx / hypopharynx
									Parotid
									Oral cavity
									Esophagus
Deganello, 2013 [11]	M	Retrospective review	18	36	16	20	RFFF	10 TEMP, 10 PMMF	Oral cavity
									Oropharynx
Fang, 2013 [12]	N	Retrospective review	18	56	20	24	RFF	PLTM	Oral cavity
					12		ALT		
Paydarfar, 2011 [23]	O	Retrospective review	20	60	33	27	RFF	SMIF	Oral cavity
Hsing, 2011 [25]	P	Retrospective review	18	100	42	58	N/A	PMMF	Oral cavity
Chan Y, 2011 [35]	Q	Prospective review	18	202	24	92	ALT	PMMF	Hypopharynx
					86		FJF		
Demirtas, 2010 [18]	R	Retrospective review	20	20	12	8	10 ALT, 2 LDF	PLDF	N/A
O’Neil, 2010 [30]	S	Retrospective review	20	114	77	37	RFFF	PMMF	N/A
Mallet, 2009 [6]	T	Retrospective review	18	70	25	45	18 RFFF, 3 LD, 3 ALT, 1 PCFF, 1 FJF	PMMF	Oral cavity
									Oropharynx
de Bree, 2007 [20]	U	Retrospective review – Matched cohort	19	80	40	40	RFFF	PMMF	Oral cavity
									Oropharynx
Smeele, 2006 [29]	V	Retrospective – Matched cohort	20	64	32	32	N/A	PMMF	Oral cavity
									Oropharynx
Chien, 2005 [26]	W	Case series	17	27	11	16	RFFF	PBFPF	Oral cavity
Chepeha, 2004 [8]	X	Retrospective review	19	179	71	108	N/A	PMMF	Oral cavity
									Oropharynx
									Hypopharynx
									Neck
									Others
Funk, 2002 [19]	Y	Case-control, matched pairs study	20	42	21	21	FTT	N/A	Oral cavity
									Oropharynx
									Hypopharynx
									Larynx
Petruzzelli, 2002 [31]	Z	Retrospective review	18	39	24	15	FTT	N/A	N/A
Amarante, 2000 [34]	Aa	Case-series	6	117	49	68	23 RFFF, 3 ORFF, 2 CFF, 5 LDF, 5 RAFF, 3 MSA, 2 PSFF, 2 ICC, 1 GOM, 3 FJF	47 PMMF, 7 PLDF, 2 PLTM, 9 TEMP, 3 MEC	Orbit
									Parotid
									Skull base
									Oropharynx
									Larynx / hypopharynx
									Mandibular
									Neck
Tsue, 1997 [24]	Ab	Retrospective review	19	53	29	24	N/A	PMMF	Oral cavity
									Oropharynx
Kroll, 1997 [13]	Ac	Retrospective review	17	178	145	33	89 RFFF, 56 RAFF	PMMF	Oral cavity
									Oropharynx
Kroll 1992 [33]	Ad	Retrospective review	18	69	30	39	RAFF	PMMF	N/A

Ref: Reference in subsequent tables

^a^MINORS score ranges from 0 to 24. A value ≥20 indicates low risk of bias

Flaps abbreviatiation: *ALT* Anterolateral thigh, *CFF* Cubital forearm flap, *DELT* Deltopectoralis, *DIEP* Deep inferior epigastric perforator flap, *FibF* Fibular free flap, *FFF* free fasciocutaneous flap, *FTT* Microvascular free tissue transfer, *FJF* Free jejunal flap, *GFMF* Gracilis free muscle flap, *GOM* Greater Omentum, *ICC* Iliac crest, *LDF* Latissimus dorsi free flap, *MEC* musculocutaneous sternocleidomastoid flap, *MSA* Muscular serratus anterior, *N/A* Not available, *ORFF* Oesteocutaneous Radial free flap, *PBFF* pedicled buccal fat flap, *PCFF* Pectoralis major free flap, *PLTM* Platysma myocutaneous island flap, *PLDF* Pedicled latissiums mucocutaneous dorsi flap, *PMMF* Pectoralis major pedicled flap, *PSFF* Parascapular free flap, *RFFF* Radial forearm free flap, *RAFF* Rectus abdominis free flap, *SCAIF* Supraclavicular artery island flap, *SMIF* Submental Island flap, *TEMP* Temporal flap, *TRPF* Trapezius flap

None of the included studies compared osseous free flaps to osseous pedicled flaps**.** All studies compared myocutaneous or fasciocutaneous flaps with the exception of two articles comparing fibular free flaps, along with other free flaps, to a variety of myocutaneous PF.

### Quality of studies

The methodological quality of each study was evaluated with the MINORS criteria [9]. (Table 1 and details in Appendix). The studies scores ranged from 6 to 20. The mean and median scores were 18.2 and 18.5 respectively. Eight studies had a MINORS score of > 20 and were considered to have low risk bias. Most studies were retrospective reviews and were deficient in categories of blinded evaluations, power calculation, and adequacy of group control. All the studies had clearly stated aims and end points were appropriate to the aim of the studies. One study had a low MINORS score of 6, but it was not excluded considering we had not established a minimum threshold for inclusion.

### Defect location

Table 1 summarizes the defects location. Twenty-three studies over the 30 included mentioned the reconstruction site, with 18 studies describing defects location and 5 studies describing tumors location. The grouping of these sites differed among the studies and variable subsite divisions were used. Both categories of flaps were not used equally to reconstruct a specific defect location within the studies, except for matching cohort studies. (Table 1 and details in Appendix).

### Demographic parameters

Mean, or median age of patients was mentioned in 19 articles. Groups were comparable in 14 studies. Five articles showed that patients were significantly younger in the FF group compared to PF (*p* <  0.05) [10–14]. Gender representation was similar between both groups in the 20 studies reporting gender, as one study [15] showed fewer males in the FF group (70.8% vs 100%, *p* <  0.05) and another [16] showed fewer males in the PF group (88% vs 32%, *p* <  0.05). (Table 2).

**Table 2:** Demographic data, preoperative risk factors and ASA class

	Total # of articles reporting (total = n)	# of articles reporting differences (total n)	Articles reporting differences	FF	PF	*p*-value
Age, mean ± SD or mean (range)	16 ^B, D, H, K, L, M, N, O, P, R, T, W, X, Z, Aa, Ac^ (*n* = 1855)	4 (*n* = 1067)	Goyal, 2017	64.0 ± 12.0	66.5 ± 12.9	0.017
			Deganello, 2013	58.2 ± 6.32	69.6 ± 6.8	< 0.01
			Fang, 2013	58.0 (25–78)	72.4 (55–80)	< 0.001
				57.2 (46–72)		< 0.001
			Kroll, 1997	56 ± 13	62 ± 12	0.0046
Age (median)	3 ^A, E, Ab^ (*n* = 601)	1 (*n* = 517)	Sinha, 2017	65.9 (57.7–74.2)	67.9 (60.3–76.8)	0.037
Age > 50 years	4 ^C, F, I, J^ (*n* = 293)	0				
Male	20 ^A, D, C, F, E, H, I, J, K, L, M, N, O, P, R, T, W, X, Aa, Ab^ (*n* = 1609)	2 (*n* = 151)	Li, 2016	17(70.83%)	17(100%)	0.043
			Zhang X, 2014	66 (88%)	31 (32.0%)	0.018
Smoking	5 ^F, K, L, T, Y^ (*n* = 300)	0		62.0%	61.8%	0.985
Prior head and neck surgery	4 ^B F X, Ab^ (*n* = 1134)	2 (*n* = 902)	Goyal, 2017	189 (32.1%)	122 (58.7%)	< 0.001
			Geiger, 2016^a^	14.0%	30.9%	0.039
Systemic diseases (CVD, HTA, DM)	1 ^N^ (*n* = 56)	1 (n = 56)	Fang, 2013	4 (20%)	20 (83.33%)	< 0.001
				3 (25%)		< 0.001
COPD	2 ^A, T^ (*n* = 587)	0				
DM	3 ^A, L, P^ (*n* = 651)	1 (*n* = 34)	Granzow, 2013	5 (31%)	0	0.02
HTA	2 ^F, L^ (*n* = 139)	0				
CAD	3 ^A, L, T^ (*n* = 621)	0				
DLP	1 ^F^ (*n* = 105)	0				
CHF	1 ^A^ (*n* = 517)	0				
aFIB	1 ^A^ (*n* = 517)	1 (*n* = 517)	Sinha, 2017	7.0%	15.0%	0.0083
Alcoholism	1 ^T^ (*n* = 70)	0				
Other cancer	1 ^T^ (*n* = 70)	0				
ASA Class I-II, n (%)	6 ^B, L, R, T, U, Y^ (*n* = 1043)	2 (*n* = 877)	Goyal, 2017	245 (41.6%)	56 (26.9%)	0.001
			de Bree, 2007	32 (80%)	37 (92.5%)	0.028
ASA Class III-IV, n (%)	6 ^B, L, R, T, U, Y^ (*n* = 1043)	2 (*n* = 877)	Goyal, 2017	343 (58.2%)	152 (73.1%)	0.001
			de Bree, 2007	8 (20%)	3 (8%)	0.028
ASA mean factor (mean ± SD)	4 ^A, H, R, Ac^ (*n* = 736)	2 (*n* = 538)	Sinha, 2017	2.6 ± 0.03	2.8 ± 0.05	0.0007
			Forner, 2016	2.3	2.4	0.05

*aFIB* atrial fibrillation, *ASA risk factor*: scored using the American Society of Anesthesiology Scale (ASA), *CAD* Cardiac artery disease, *CHF* Chronic heart failure, *COPD* Chronic obstructive pulmonary disease, *CVD* Cardiovascular disease, *DM* Diabetes mellitus, *DLP* Dyslipidemia, *HTA* Hypertension

### Preoperative risk factors

Table 2 shows the preoperative risk factors for the FF and PF groups. Both were comparable for the incidence of smoking, chronic obstructive pulmonary disease (COPD), hypertension (HTA), cardiac atherosclerosis disease (CAD), dyslipidemia (DLP), chronic heart failure (CHF), alcoholism and the incidence of other cancer.

Prior head and neck surgery was reported in 4 studies. Two studies [10, 17] found there was a lower proportion of patients who had prior head and neck surgery in the FF group compared to the PF group (32% vs 59 and 14% vs 31%, *p* <  0.05). One study [12] reporting the incidence of preoperative systemic disease showed a lower regrouped incidence of diabetes, cardiovascular disease and hypertension in the FF group (25% vs 83%, *p* <  0.05). In contrast, another study [7] demonstrated a higher incidence of diabetes mellitus in the FF group compared to PF (31% vs 0, *p* <  0.05). The only study reporting the incidence of atrial fibrillation [14] showed less patient with atrial fibrillation in the FF group compared to PF (7% vs 15%, *p* <  0.05).

### American Society of Anesthesiologists (ASA) classification

Six studies reported ASA class. Among them, four showed similar ASA classes [6, 7, 18, 19]. Two studies showed a significant difference in ASA class between FF and PF groups with contrasting results. Goyal et al. [10] showed a higher proportion of ASA class I-II in the FF group (41.6% vs 26.9%, *p* <  0.05) and a lower proportion of ASA class III-IV in the FF group (58.2% vs 73.1%, *p* <  0.05) compared to PF. 

In contrast, de Bree et al. [20] demonstrated a lower proportion of patients with ASA class I-II in the FF group (80% vs 92.5%, *p* <  0.05) and a higher proportion of ASA class III-IV in the FF group (20% vs 8%, *p* <  0.05). (Table 2.)

### Prior radiation or chemotherapy

Exposure to prior head and neck radiation therapy was mentioned in 11 articles, and was comparable between the FF and PF groups in 9 of them [5–8, 13, 17, 21–23]. (Table 3) Two articles [10, 24] showed a significantly lower proportion of prior radiotherapy with FF reconstruction compared to PF (46% vs 62 and 28% vs 54% *p* <  0.05). No difference was seen in the incidence of prior chemotherapy between FF and PF, as it was reported in five studies [5, 7, 17, 23, 24]. The incidence of adjuvant chemoradiotherapy after surgery was higher in the FF group in one study (48% vs 44%, *p* < 0.05) [23].

**Table 3:** Staging and treatment data

	Total # of articles reporting (total = n)	# of articles reporting differences (total n)	Articles reporting difference	FF	PF	*p*-value
Prior radiation	11 ^B, D, E, F, K, L, O, T, X, Ab, Ac^ (*n* = 1628)	2 (*n* = 850)	Goyal, 2017	272 (46.2%)	130 (62.5%)	< 0.001
			Tsue, 1997	8 (28%)	13 (54%)	0.05
Prior chemotherapy	5 ^D, K, L, O, Ab^ (*n* = 268)	0				
T1	8 ^J, K, M, N, O, P, W, Y^ (*n* = 480)	1 (*n* = 36)	Deganello, 2013	0	4 (20%)^#^	< 0.01
T2	8 ^J, K, M, N, O, P, W, Y^ (*n* = 480)	1 (*n* = 36)	Deganello, 2013	7 (43.8%)^#^	5 (25%)^#^	< 0.01
T3	8 ^J, K, M, N, O, P, W, Y^ (*n* = 480)	1 (*n* = 36)	Deganello, 2013	8 (50%)	8 (40%)	< 0.01
T4	8 ^J, K, M, N, O, P, W, Y^ (*n* = 480)	1 (*n* = 36)	Deganello, 2013	1 (6.25%)	3 (15%)	< 0.01
T1-T2	4 ^C, H, T, Ab^ (*n* = 185)	0				
T3-T4	4 ^C, H, T, Ab^ (n = 185)	0				
Stage I-II	1 ^X^ (*n* = 179)	0				
Stage III-IV	1 ^X^ (*n* = 179)	0				
Surg + chemoradio	6 ^C, N, O, P, U^ (*n* = 516)	1 (*n* = 60)	Paydarfar, 2011	16 (48.48%)^#^	12 (44.44%)^#^	0.03
Surg + radio	4 ^J, O, P, X^ (*n* = 449)	0				
Tumor stage	1^Ac^ (*n* = 178)	0				
Tumor recurrence	1^Ac^ (*n* = 178)	0				

Surg + chemoradio: Surgical resection and adjuvant chemoradiotherapy

Surg + radio: Surgical resection and adjuvant radiotherapy

# Percentage calculated relying on the data presented. Percentage not provided by the article

Bold = Statistically significant, *p*-value ≤ 0.05

### Tumor staging

Tumor stages were compared in 13 studies; T stage or global staging was reported (Table 3). No significant difference in cancer staging was found in the other 12 studies [6, 11, 12, 15, 16, 19, 22–27]. A study comparing RFFF to PMMF and temporalis flap [11] showed a lower proportion of T1 and T4 in the FF group (T1: 0% vs 20%; T4: 6.25% vs 15%, *p* < 0.05) as well as a higher proportion of T2 and T3 in the FF group (T2: 43.8% vs 25%; T3:50% vs 40%, *p* < 0.05) when compared to PF. 

### Operative time

Nineteen studies compared the operative time between both reconstruction techniques. All showed that FF was associated with a longer operating time than PF. This difference was statistically significant in 14 studies (Table 4) [5, 7, 10, 13–16, 20, 21, 23, 24, 27–29].

**Table 4:** OR time, Hospital and ICU length and hospital cost

	Total # of articles reporting (total = n)	# of articles reporting differences (total n)	Articles reporting difference	FF	PF	*p*-value
OR time, min (mean ± SD)	12 ^A, B, C, E, H, I, L, M, O, R, U, Ab^ (*n* = 1727)	9 (*n* = 1634)	Sinha, 2017	421.4 ± 4.4	332.7 ± 10.7	0.0001
			Goyal, 2017	427.2 ± 92.3	310.8 ± 125.0	0.001
			Li, 2016	405 ± 107	365 ± 48	< 0.05
			Howard, 2016	683 (575–979)	544 (396–700)	0.00817
			Forner, 2016	552	347	< 0.05
			Granzow, 2013	816.3 ± 148.9	587.9 ± 130.5	0.0002
			Paydarfar, 2011	780	506.4	0.001
			de Bree, 2007	692	462	< 0.005
			Tsue, 1997	684 ± 16	666 ± 20	0.003
OR time, hour (mean ± SD)	6 ^D, N, T, V, Ac, Ad^ (*n* = 474)	4 (*n* = 384)	Kozin, 2016	8.1	6.7	0.002
			Mallet, 2009	7.01 ± 1.19)	4.19 ± 0.57	< 0.001
			Smeele, 2006	12.5 ± 1.9	9.9 ± 1.5	< 0.0001
			Kroll, 1997	10.49 ± 2.06	9.39 ± 2.59	0.029
OR time of > 600 min	1 ^J^ (*n* = 110)	1 (*n* = 110)	Zhang X, 2014	59 (74.68%)	3 (9.68%)	0.001
Hospit length, days (mean ± SD)	17 ^D, E, H, I, L, M, O, R, S, T, U, V, X, Z, Ab, Ac, Ad^ (*n* = 1104)	7 (*n* = 634)	Howard, 2016	9.8 (7–22)	4.75 (2–14)	0.004
			Zhang S, 2015	17 ± 2.5	12 ± 1.7	< 0.05
			Paydarfar, 2011	14.0	10.6	0.008
			de Bree, 2007	24	28	0.005
			Chepeha, 2004	12	14	0.006
			Kroll, 1997	13.2 ± 5.4	19.8 ± 11.5	0.003
			Kroll, 1992	11.3	21.2	0.003
ICU length, days (mean ± SD)	4 ^H, L, V, Ab^ (*n* = 172)	1 (*n* = 34)	Granzow, 2013	5.6 (4–9)	1.8 (0–5)	0.0001
Hospital cost	9 ^D, H, M, R, U, V, Z, Ab, Ac^ (*n* = 563)	4 (*n* = 339)	Kozin, 2016	SCAIF 32% less expensive than FTT		0.0001
			Deganello, 2013	22,924	19,872	0.043
			Tsue, 1997	50,026 ± 4340	38,246 ± 1440	0.003
			Kroll, 1997	28,460 ± 8435	40,992 ± 1958	0.001

### Hospitalization and ICU length of stay

Seventeen studies compared the duration of hospital stay. (Table 4). Ten studies showed a similar hospitalization stay with FF compared to PF [5–7, 11, 18, 24, 27, 29–31]. However, when FF were compared to SMIF and SCAIF specifically the results differed. FF patients had a longer hospitalization stay than SMIF patients for skull base (9.8 days vs 4.75, *p* < 0.05) [21] and oral cavity (14.0 days vs 10.6, *p* < 0.05) defects [23]. SCAIF patients had a shorter length of stay than FF patients for oral cavity defects (12 ± 1.7 vs 17 ± 2.5, *p* < 0.05) [32]. On the other hand, four studies [8, 13, 20, 33] showed a shorter hospitalization stay with FF compared to PMMF (*p* < 0.05).

Four studies assessed the ICU length of stay [7, 24, 27, 29]. One study comparing FF to SCAIF for larynx/pharynx reconstruction [7] concluded in a longer stay for FF reconstruction (*p* < 0.05).

### Cost

Nine studies compared hospital costs between FF and PF [5, 11, 13, 18, 24, 27, 29, 31]. As reported by three studies, FF was associated with a significantly higher cost compared to PF. Of these, Kozin et al. [5] showed that SCAIF was 32% less expensive than FF for total laryngectomy, parotid/temporal bone, and cutaneous defect reconstruction. Reconstruction for oral cavity and oropharynx were also less expensive with PMMF compared to FF. (38,246$ vs 50,026, *p* < 0.05) [24]. A study comparing temporal flap (TEMP) and PMMF reconstruction to RFFF for oral cavity and oropharyngeal defects to RFFF led to similar findings (19,872$ vs 22,924$, *p* < 0.05) [11]. In contrast, one study [13] showed a lower hospital cost with FF compared to PMMF for the reconstruction of oral and oropharyngeal defects. (28,460 ± 8435 vs 40,992 ± 1958, *p* < 0.05) (Table 4).

### Post-operative complications

Articles differed in the definitions of their studied complications. (Table 5). For example, some studies grouped recipient and donor site complications, as other separated them. Some studies were less specific and only reported the incidence of any complications. Others were selectively reporting the incidence of infection, fistula, abscess, dehiscence, hematoma, and others. Articles and results were grouped according to the definition of their studied complications.

**Table 5:** Post-operative complications and outcomes

	Total # of articles reporting (total = n)	# of articles reporting differences (total n)	Articles reporting difference	FF	PF	*p*-value
Any complications	7 ^F, J, K, L, O, Aa, Ad^ (*n* = 544)	3 (*n* = 284)	Geiger, 2016	68.0%	36.4%	0.001
			Zhang X, 2014	13 (16.46%)	14 (45.16%)	0.002
			Kroll, 1992	4 (13%)	17 (44%)	0.0145
Infection	3 ^F, L, T^ (*n* = 209)	0				
Recipient site infection	6 ^B, D, O, S, T, X^ (*n* = 1292)	1 (*n* = 179)	Chepeha, 2004	2 (3%)	18 (17%)	< 0.004
Donor site infection	3 ^B, D, S^ (*n* = 983)	0				
Donor site morbidity	1 ^Q^ (*n* = 202)	0				
Fistula	11 ^B, F, K, O, Q, S, R, T, V, X, Aa^ (*n* = 1777)	2 (*n* = 902)	Goyal, 2017	18 (3.1%)	17 (8.2%)	0.005
			Geiger, 2016	22.0%	7.3%	0.039
Abscess	1 ^F^ (*n* = 105)	0				
Dehiscence recipient or donor site	4 ^F, K, S, V^ (*n* = 332)	1 (*n* = 105)	Geiger, 2016	44.0%	23.6%	0.029
Dehiscence recipient site	5 ^D, L, O, V, X^ (*n* = 409)	1 (*n* = 179)	Chepeha, 2004	0	11 (10%)	< 0.008
Dehiscence donor site	8 ^D, I, L, O, V, K, L, R^ (*n* = 370)	0				
Hematoma	2 ^X, S^ (*n* = 293)	0				
Hematoma Donor site	2 ^E, V^ (*n* = 95)	0				
Hematoma recipient site	2 ^O, V^ (*n* = 124)	0				
Partial flap necrosis	6 ^I, O, V, X, Aa, Ad^ (*n* = 526)	1 (*n* = 179)	Chepeha, 2004	2 (2.82%)^#^	12 (11%)^#^	< 0.006
Total flap necrosis	2 ^V, Aa^ (*n* = 181)	0				
Partial or total flap necrosis	1 ^T^ (*n* = 70)	1 (*n* = 70)	Mallet, 2009	1 (4%)	14 (31%)	0.02
Osteonecrosis	2^B, F^ (*n* = 902)	1 (*n* = 105)	Geiger, 2016	24.0%	3.6%	0.007
Deep Vein Thrombosis (Inferious member)	2 ^A, S^ (*n* = 631)	0				
Venous obstruction (At site)	1 ^O^ (*n* = 60)	0				
Late anastomotic stricture	1^Q^ (*n* = 202)	0				
Operative revision surgery	8 ^E, F, L, O, S, R, X, Aa^ (*n* = 660)	2 (*n* = 136)	Howard, 2016	1.6 (1–3)	0.6 (0–1)	< 0.00001
			Geiger, 2016	34%	9.1%	0.003
Flap failure	8 ^E, I, K, L, O, T, U, V^ (*n* = 425)	1 (*n* = 70)	Mallet, 2009	1 (4%)	14 (31%)	0.02
Mortality at 30 days	2 (*n* = 228)^K, X^					
Mortality at 1-year	2 (*n* = 76)^L, Y^					
Mortality at 2-year	1 (*n* = 80)^U^					

# Percentage not provided by the original article, calculated by the authors from the data presented

Seven articles reported the incidence of “any complications” [7, 16, 17, 22, 23, 33, 34]. One article [17] showed that FF was associated with a higher incidence of any complication (68.0% vs 36.4%, *p* < 0.05). This article included various types of flaps in both FF and PF groups. Two studies [16, 33] showed the opposite with a lower incidence of “any complication” in the FF group compared to PF. Of those, Zhang X et al. [16] showed a significantly lower rate of complications in the FF group compared to PMMF. (16.5% vs 45.2%, *p* < 0.05).

Infections at large, recipient site infection and donor site infection were reported in some studies. In one study [8], the rate of infection at the recipient site was lower in the FF group compared to the PMMF group (3% vs 17%, *p* < 0.05).

Two studies showed significant differences in the incidence of fistula. Goyal et al. [10] showed a lower rate of fistula in the FF group compared to PF (3.1% vs 8.2%, *p* < 0.05). The exact defect location was not specified in this study including multiple reconstruction sites, i.e. skull base, sinonasal cavities, oral cavity, and larynx. However, in a study focusing on intraoperative brachytherapy [17], the rate of fistula was higher in the FF group (22% vs 7.3%, *p* < 0.05). Neither the defect location nor the exact type of flaps was mentioned in this study.

The incidence of dehiscence either at the recipient, donor, or recipient and/or donor sites was reported by several studies. Dehiscence at “recipient and/or donor” site was higher with FF reconstruction compared to PF, according to Geiger et al. (44% vs 23.6%, *p* < 0.05) [17].

Dehiscence at recipient site was lower in FF group compared to PMMF in one study (0 vs 10%, *p* < 0.05) [8]. As for dehiscence at the donor site, no significant difference was observed between FF and PF in the eight studies reporting this complication [5, 7, 18, 22, 23, 29, 32].

The incidence of hematomas either at the recipient, donor, or recipient and/or donor site was analyzed by very few studies [8, 21, 23, 29, 30], without statistically significant differences between both techniques.

One study [8] showed a lower incidence of partial flap necrosis with FF reconstruction compared to PMMF (2.8% vs 11%, *p* < 0.05) for various defect locations (oral cavity, oropharynx, hypopharynx, neck, and others). The same was revealed by the Mallet et al. [6] study where “partial or total flap” necrosis was higher with PMMF for oral tongue and base of tongue reconstruction (4% vs 31%, *p* < 0.05).

### Post-operative outcomes

Operative revision surgery was significantly higher in the FF group in two studies [21] (Table 5). One compared FF to SMIF (1.6 vs 0.6, *p* < 0.05) for lateral skull base defects [21] and the other compared various FF to PF (34% vs 9.1%, *p* < 0.05) without specifying the defect location [17]. Although not being statistically significant, six other studies also showed a higher occurrence of revision with FF reconstruction [7, 8, 18, 23, 29, 30, 34]. One study [6] showed that flap failure was more frequent with PMMF compared to FF (4% vs 31%, *p* < 0.05) for oral tongue and base of tongue reconstruction. No difference between both groups was reported for mortality at 30 days [8, 22], at 1 year [7, 19] and at 2 years [20].

### Quality of life

Table 6 shows the quality of life of patients after surgical reconstruction with either FF or PF. The University of Washington Quality of Life Questionnaire (UW-QOL), including 14 items, was used by three studies [15, 16, 25] to measure the quality of life after surgical reconstruction with either FF or PMMF. Differences were seen in speech. Zhang X. et al. [16] showed a lower quality of speech with FF (57.5 ± 20.1 vs 76.1 ± 13.3, *p* < 0.05) with a mean follow-up of 5.9 years. Hsing et al. showed a better quality of speech with FF compared to PMMF (66.7 ± 27.2 vs 44.7 ± 35.0, *p* < 0.05) from data of patients operated 2 to > 10 years earlier.

**Table 6:** Quality of Life data

		Article	FF	PF	*p*-value
UW-QOL Global		Li, 2016^#^	55.14 ± 9.24	54.36 ± 8.13	0.965
		Zhang X, 2014^$^	70.5 ± 16.7	67.3 ± 12.9	0.860
		Hsing, 2011^&^	66.0 ± 18.5	57.8 ± 18.2	0.090
UW-QOL: Pain		Li, 2016	71.63 ± 9.91	72.94 ± 11.13	0.751
		Zhang X, 2014	86.2 ± 10.8	89.9 ± 11.4	0.425
		Hsing, 2011	76.8 ± 23.0	68.1 ± 27.2	0.138
UW-QOL: Swallowing		Li, 2016	44.00 ± 16.27	43.78 ± 4.95	0.741
		Zhang X, 2014	49.4 ± 14.7	51.3 ± 21.7	0.840
		Hsing, 2011	49.3 ± 37.2	48.6 ± 32.7	0.962
UW-QOL: Chewing		Li, 2016	42.45 ± 6.15	43.43 ± 12.37	0.817
		Zhang X, 2014	52.6 ± 17.1	59.4 ± 12.9	0.498
		Hsing, 2011	34.5 ± 39.0	33.6 ± 36.7	0.973
UW-QOL: Speech		Li, 2016	51.27 ± 11.24	52.63 ± 12.43	0.461
		Zhang X, 2014	57.5 ± 20.1	76.1 ± 13.3	**0.017**
		Hsing, 2011	66.7 ± 27.2	44.7 ± 35.0	**0.002**
UW-QOL: Apparence		Li, 2016	57.47 ± 11.44	68.54 ± 13.24	**0.0001**
		Zhang X, 2014	76.4 ± 18.6	70.3 ± 17.1	0.308
		Hsing, 2011	67.3 ± 25.0	69.8 ± 25.5	0.535
UW-QOL: Activity		Li, 2016	64.23 ± 9.52	63.73 ± 8.41	0.641
		Zhang X, 2014	71.9 ± 11.5	74.8 ± 10.2	0.710
		Hsing, 2011	67.9 ± 24.2	66.8 ± 27.9	0.760
UW-QOL: Recreation		Li, 2016	66.59 ± 11.62	67.26 ± 9.23	0.445
		Zhang X, 2014	72.1 ± 10.2	78.9 ± 11.2	0.590
		Hsing, 2011	69.1 ± 32.6	62.5 ± 32.2	0.221
UW-QOL: Shoulder		Li, 2016	61.52 ± 7.83	54.65 ± 11.24	**0.0001**
		Zhang X, 2014	87.1 ± 14.4	65.6 ± 20.0	**< 0.001**
		Hsing, 2011	81.4 ± 14.7	50.5 ± 29.8	**< 0.001**
UW-QOL: Taste		Li, 2016	50.91 ± 10.64	51.24 ± 11.23	0.673
		Zhang X, 2014	48.4 (18.3)	52.9 (19.6)	0.713
		Hsing, 2011	55.0 ± 43.2	45.9 ± 39.6	0.226
UW-QOL: Salive		Li, 2016	45.48 ± 16.92	44.17 ± 12.78	0.723
		Zhang X, 2014	70.9 ± 9.5	72.3 ± 23.1	0.813
		Hsing, 2011	71.7 ± 34.8	73.8 ± 28.1	0.964
UW-QOL: Mood		Li, 2016	69.94 ± 9.51	68.31 ± 14.72	0.474
		Zhang X, 2014	76.0 ± 14.7	71.6 ± 18.8	0.114
		Hsing, 2011	76.2 ± 24.7	60.8 ± 32.8	**0.022**
UW-QOL: Anxiety		Li, 2016	70.57 ± 15.11	72.55 ± 15.19	0.219
		Zhang X, 2014	78.5 ± 9.64	86.4 ± 17.5	0.775
		Hsing, 2011	75.9 ± 26.3	68.9 ± 33.9	0.423
UW-QOL: Composite score		Hsing, 2011	66.0 ± 18.5	57.8 ± 18.2	0.090
Speech	Excellent	Zhang S, 2015^§^	12 (80.0%)^#^	11 (91.7%)^#^	0.62
	Good		3 (20%)	1 (8.3%)	
	Poor		0	0	
	Always understandable	O’Neil, 201	17 (53.1)	4 (22.2)	**0.014**
	Usually understandable		14 (43.8)	9 (50.0)	
	Difficult to understand		1 (3.1)	5 (27.8)	
Swallowing full/regular diet at follow-up (vs soft, liquid) n (%)		Zhang S, 2015^§^	13 (86.7%)^#^	10 (83.3%)^#^	1.00
		Paydarfar, 2011^%^	19	20	0.60
		Chan Y, 2011*	8 (38.2%)	24 (35.8%)	ND
			52 (61.9%)		ND
		O’Neil, 2010**	17 (59.4%)	6 (33.3%)	0.202
		Tsue, 1997***	8 (34%)	4 (17%)	**0.02**
Preoperative mouth-open width distance (mean) cm		Fang, 2013	1.5–6.2 (4.6)	1.2–6.2 (4.8)	ND
			0.9–6.0 (3.5)		ND
		Chien, 2005	6.3–3.5 (5.7)	6.1–2.5 (5.1)	0.384
Postoperative mouth-open width		Fang, 2013	1.4–5.8 (4.3)	1.1–4.7 (3.2)	ND
			0.8–5.8 (3.3)		ND
		Chien, 2005	5.9–3.2 (5.2)	5.6–1.6 (3.6)	0.384
Mouth-open width change (%)		Fang, 2013	4.0–9.1%	8.3–47.5%	**< 0.001**
			3.3–11.1%		**<0.001**
		Chien, 2005	4.8–9.8%	5–45.5%	**< 0.001**
G-tube at 6 months postoperatively		Smeele, 2006	21.8%	34.3%	NS
G-tube dependence, n (%)		Chepeha, 2004	10 (16%)	40 (42%)	**0.001**
Feeding tube for >21 days		Mallet, 2009	8 (36%)	17 (42%)	0.84
Feeding tube at discharge		Tsue, 1997	20 (69%)	20 (83%)	NS
Feeding tube at follow up^e^			11 (39%)	17 (85%)	**0.002**

# Follow up ranging from 13 to 108 months

$ Mean-follow up = 5.9 years

& Follow up ranging from 2 to >10 years

§ Follow-up = 6 months

% at most recent follow-up

* Regular PO follow-up, median follow-up period was 82 months

** Follow-up period not mention

*** Median follow-up was 298 days

# Percentage calculated relying on the data presented. Percentage not provided by the article

Bold = Statistically significant, *p*-value ≤ 0.05

Speech quality was also specifically assessed by two other studies not using the UW-QOL. O’Neil et al. [30] found a difference in speech quality (*p* < 0.05), with RFFF patients being more often “always understandable” than PMMF patients (53.1%vs 22.2%, follow-up period not mentioned). Additionally, Zhang S. et al. [32] graded the speech quality as excellent, good or poor, and found no difference in the speech quality of reconstruction with either FF or SCAIF flaps 6 months after the surgery.

Shoulder function, evaluated with UW-QOL, was significantly better in the FF group compared to the PMMF group in all three studies [15, 16, 25]. Follow-up time was ranging from 1 to over 10 years. One study [25] showed that FF was associated with a better mood compared to PMMF (76.2 ± 24.7 vs 60.8 ± 32.8, *p* < 0.05).

In addition, looking at studies using the UW-QOL, FF and PMMF scored similarly on global quality of life, pain, swallowing, chewing, speech, activity, recreation, taste, saliva, anxiety and composite score [15, 16, 25].

Recovery to a normal diet was reported in five studies [23, 24, 30, 32, 35]. According to one study [24], the incidence was higher in the FF group compared to the PMMF for reconstruction of oral or base of tongue defects (34% vs 17%, *p* < 0.05).

Preoperative and postoperative mouth opening were reported by two studies, one comparing RFFF and ALT to platysma myoctuaneous island flap (PMIF) [12] and the other comparing RFFF to pedicled buccal fat pad flap [26]. Mouth opening was similar between FF and PF groups.

The incidence of feeding tube dependence was reported by some studies and different postoperative timepoints were evaluated. One study [8] showed a lower incidence of feeding tube dependence in the FF group compared to PMMF (16% vs 42%, *p* < 0.05) for reconstruction of various defects. The FF group was also associated with a lower rate of incidence of feeding tube at follow up, with a median follow-up of 298 days, compared to PMMF (39% vs 85%, *p* < 0.05) for oral cavity and oropharynx reconstruction [24]. Feeding tube dependence at 21 days [6] and at discharge [24] was similar between the PF and FF groups in two studies.

## Discussion

Choosing between FF and PF in head and neck reconstruction is a challenge for some defects, especially with the recent resurgence of PF and their expanding indications. In this era of economic awareness in the healthcare system, use of microvascular reconstruction needs to be justified if other comparable and less expensive alternatives are available. The present study aimed to review the literature comparing FF to PF for reconstruction of oncologic head and neck defects and determine the relative benefits and drawbacks of both flap types. To our knowledge, this is the first systematic review of studies comparing the postoperative complications and outcomes of FF and PF for head and neck reconstruction of oncologic defects.

The major findings of the present study are that: (a) FF was associated with a longer operating time and, in general, a higher cost compared to PF, including compared to SCAIF. (b) FF was associated with a lower hospitalization stay compared to PMMF, but a higher hospitalization stays when compared to SCAIF and SMIF. (c) Recipient site morbidity was lower with FF reconstruction compared to PMMF, including a lower incidence of infection, dehiscence, and necrosis. The incidence of hematoma and fistula were equivocal. (d) Donor site morbidity was equivocal between FF and PF reconstruction, with no distinction in the rate of infection, dehiscence, and hematoma. (e) Revision surgery was higher with FF reconstruction compared to PF and SMIF. (f) Speech quality was better with FF than with PMMF for oral cavity defects, and FF and PMMF scored similarly on global quality of life, pain, swallowing, chewing, speech, activity, recreation, taste, saliva, anxiety and composite score.

Those conclusions are drawn from retrospective studies lacking methodological homogeneity, thus limiting a truly valid comparison between FF and PF reconstruction. The main issues that need to be further address are the inherent differences among the studied groups in term of patients’ preoperative characteristics and defect locations. The findings of the studies included in this review can result from surgeon’s bias itself opting for either a FF or a PF based on patient’s characteristics and considering it more suitable for a certain location. In fact, patients in the FF group were younger than patients in the PF group with more than a 10-year age difference noted in some studies [11, 12]. Distal extremity reconstruction donor sites are thought to be affected by the patients’ health status and age in relation to the condition of peripheral vessels. However, according to several studies age is not considered a risk factor for FF failure [36]. FF reconstruction was also considered with favorable long-term outcomes in patients of 90 years old in a study by Wester et al. [37].

Overall, only a minority of studies showed significant differences in the preoperative characteristics of FF and PF groups. The patients characteristics and T stage were similar between FF and PF groups in most of the studies with a few exceptions. In those, some even showed opposite findings, as it is the case for the ASA class and the incidence of diabetes mellitus [7, 10, 12, 20]. Thus, in front of a majority of studies with similar baseline characteristics between the PF and FF groups, we could extrapolate with caution that the intrinsic flaps characteristics have an essential contribution to the surgical outcomes depicted in these studies.

A unanimous finding among all studies in this review was the longer operative (OR) time necessary for FF reconstruction which was frequently explained by the microvascular anastomosis. Interestingly, four distinct articles mentioned longer hospitalization time for PF when compared to PMMF [8, 13, 20, 33]. The higher complication rate in PMMF and the poorer patients’ preoperative health status in two of those studies may explain this finding [25, 33]. In contrast, SMIF and SCAIF showed a shorter hospitalization and ICU length of stay when compared to FF in similar patients groups [7, 21, 23, 32].

Cost analysis favour PF over FF in a study focusing on the SCAIF [5]. The study by Forner et al. also showed a favorable cost-analysis for SMIF over RFFF but no statistical analysis was provided to be able to conclude on a significant difference [27]. Conclusions on the relative cost of PMMF are harder to draw because studies are showing divergent results [11, 13, 24]. The differences in costs for the PMMF between studies can be explained by the different indications for the use of this flap by the authors. In an era of limited resources and increased attention to health economics, cost analysis studies should be encouraged.

Studies demonstrating significant differences in complication rates were all specifically comparing PMMF to FF. In fact, there was a higher incidence of overall complications, recipient site infection, dehiscence of recipient site, necrosis and flap failure with PMMF reconstruction in all articles except one. Geiger et al. [17] presented different results with regards to fistula, dehiscence and osteonecrosis rates. It is important to note, however, that the authors compared RFFF to PMMF only in the presence of intraoperative brachytherapy implants. These implants, which supplied high doses of radiation, may have led to direct tissue damage in the thinner free flaps, subsequently leading to a higher risk of fistula and dehiscence. The authors themselves associated the lower complication rate of the PMMF group to their increased bulk.

When comparing ALT to SMIF, Howard et al. [21] showed a higher complication rate as well as higher operative revision rates when using the ALT. Similarly, Zhang et al. [23] demonstrated higher rates of donor site complications in the RFFF when compared to SCAIF. Paydarfar et al. [23] demonstrated higher recipient and donor site complications when comparing RFFF to SMIF (no *p*-values were available); this latter flap has previously been cited as having a low donor site morbidity in another study [38].

PMMF was associated with poorer QoL outcomes when compared to FF [15, 16, 25, 32]. Tsue and al [24]. even demonstrated a lower capacity to progress to a regular diet following oral cavity and oropharyngeal reconstruction. This was corroborated by Chepeha et al. [8] who showed a higher incidence of gastrostomy tube dependence after PMMF which they attributed to the flap’s downward pull, small size, a limited axis of rotation and inability to fold.

Thereby, PMMF seem to be inferior to FF or other pedicled flaps on many different levels. However, we must remember that higher ASA classes were more common in the PMMF groups representing a considerable bias in the literature. It is our opinion that PMMF should still be considered a reliable and useful flap, especially in a salvage surgery setting.

The present review did not allow us to find any comparative study between osseous or composite FF and osseous or composite PF. Composite head and neck defects have previously been reconstructed with PF including a bony component such as the pectoralis major osteomyocutaneous flap with rib or sternum, the sternocleidomastoid flap with part of the clavicle, or the trapezius flap with the scapular spine. These flaps did not withstand the test of time because of their lack of robustness, reliability and versatility in comparison to their homologue free flaps [39–43]. The more recent SMIF has also been used as a composite flap for mandible, maxilla and orbital defects reconstruction [10]. Yet, its role in osseous reconstruction remains to be defined in an era dominated by FF. Despite the lack of comparative studies, we can safely state that FF are superior to PF for bony reconstructions, especially in radiated patients.

This review suggests that SMIF and SCAIF can be considered reasonable alternatives to free flaps for the reconstruction of head and neck tissue defects given the similar functional outcomes and better performance in OR time, hospitalization/ICU length, and cost. Some articles described their use more frequently in higher ASA classes which further highlights their utility. They can usually be closed primarily and do not typically require skin grafting [38]. Furthermore, SMIF has also been cited as having superior color matching for cervicofacial skin defects [44]. Nonetheless, SMIF and SCAIF are not suitable for all head and neck defects. Patients with previous history of radiation or ipsilateral neck dissection are not optimal candidates [21]. Additionally, reconstruction of the midface or upper face can sometimes be limited by the length of their respective pedicles. Finally, the use of SMIF in patients requiring level I neck dissection is still debated [21], as its oncological safety and the potential risk to transfer cervical neoplastic cells to the recipient site is controversial in the literature [45]. However, recurrence is thought to be due to the aggressively of the resected tumor than the flap itself [46]. A careful flap dissection, at the subplatysmal plane, after completing the neck dissection, helps minimize the risk of tumor spread [47]. The SMIF is a reliable reconstruction technique if level 1, A and B, nodes are thoroughly removed, as supported by Howard et al. 11-years case-series study, where no recurrences related to the SMIF transfer of metastatic tissue were noted [21]. Still, SMIF should not be performed in the presence of clinical or radiographic evidence of level 1 cervical lymph node disease [48].

## Limitation

Of the thirty studies reviewed, some do not specify the primary tumor location and consequently the defect site. Many articles did not mention the specific flaps used and lacked standard definitions for post-operative complications and outcomes. Furthermore, retained articles were for the majority retrospective studies and comprised risk bias, as assessed by the MINORS criteria. These factors limited the authors ability to analyze specific discordant results between articles and to draw robust conclusions from this systematic review.

## Conclusion

The articles included in this review are lacking of methodological homogeneity. Their retrospective nature and the inherent disparities in term of preoperative characteristics between the groups in some studies are limiting. Although the conclusions should be interpreted with caution, it is safe to assume that free flaps are an excellent choice for reconstruction in relatively healthy subjects with low ASA classes. It appears that FF are superior to the PMMF for several postoperative outcomes. However, other pedicled flaps such as the SMIF and SCAIF compare favorably to FF for some specific indications achieving similar outcomes at a lower cost.

## Appendix

**Table 7:** Methodological Quality of Included Studies by MINORS Criteria

Study, Year	Type of Study	Score	Methodological items for non-randomized studies								*Additional criteria in the case of comparative study*			
			Clearly Stated Aim	Inclusion of Consecutive Patients	Prospective Collection of Data	Endpoints Appropriate to Aim of Study	Unbiased Assessment of Study Endpoint	Appropriate Follow-up Period to Aim of Study	Loss to Follow-up <5%	Prospective Calculation of Study Size	An adequate control group	Contemporary groups	Baseline equivalence of groups	Adequate statistical analyses
Sinha, 2017	Retrospective review	20	2	2	2	2	0	2	2	2	2	2	0	2
Goyal, 2017	Retrospective review	18	2	2	2	2	0	2	2	0	2	2	0	2
Li, 2016	Retrospective review	19	2	2	2	2	0	2	2	0	2	2	1	2
Kozin, 2016	Retrospective review	20	2	2	2	2	0	2	2	0	2	2	2	2
Howard, 2016	Retrospective review	18	2	2	2	2	0	2	2	0	2	2	0	2
Geiger, 2016	Retrospective review	20	2	2	2	2	0	2	2	0	2	2	2	2
Gao, 2016	Retrospective review	16	2	0	2	2	0	2	2	0	2	2	0	2
Forner, 2016	Retrospective review	20	2	2	2	2	0	2	2	0	2	2	2	2
Zhang S, 2015	Retrospective review	19	2	2	2	2	0	2	2	0	2	2	1	2
Zhang X, 2014	Retrospective review	19	2	2	2	2	0	2	1	0	2	2	2	2
Jing, 2014	Retrospective review	17	1	2	2	2	0	2	2	0	2	2	0	2
Granzow, 2013	Retrospective review	18	1	2	2	2	0	2	2	0	2	2	1	2
Deganello, 2013	Retrospective review	18	2	2	2	2	0	2	2	0	2	2	0	2
Fang, 2013	Retrospective review	18	2	2	2	2	0	2	2	0	2	2	0	2
Paydarfar, 2011	Retrospective review	20	2	2	2	2	0	2	2	0	2	2	2	2
Hsing, 2011	Retrospective review	18	2	2	2	2	0	2	2	0	2	2	0	2
Chan Y, 2011	Prospective	18	2	2	2	2	0	2	2	0	2	2	0	2
Dermirtas, 2010	Retrospective review	20	2	2	2	2	0	2	2	0	2	2	2	2
O’Neil, 2010	Retrospective review	20	2	2	2	2	0	1	2	2	2	2	1	2
Mallet, 2009	Retrospective review	18	2	2	2	2	0	1	2	0	2	2	1	2
de Bree, 2007	Retrospective review – Matched cohort	19	2	2	2	2	0	2	1	0	2	2	2	2
Smeele, 2006	Retrospective review – Matched cohort	20	2	2	2	2	0	2	2	0	2	2	2	2
Chien, 2005	Case series	17	2	2	2	2	0	1	2	0	2	2	0	2
Chepeha, 2004	Retrospective review	19	2	2	2	2	0	1	2	0	2	2	2	2
Funk, 2002	Case-control study	20	2	2	2	2	0	2	2	0	2	2	2	2
Petruzzelli, 2002	Retrospective review	18	2	2	2	2	0	2	2	0	2	2	0	2
Amarante, 2000	Case-series	6	1	0	2	1	0	0	0	0	2	0	0	0
Tsue, 1997	Retrospective review	19	2	2	2	2	0	2	2	0	2	2	1	2
Kroll, 1997	Retrospective review	17	2	2	2	2	0	2	2	0	2	2	1	0
Kroll 1992	Retrospective review	18	2	2	2	2	0	2	2	0	2	2	0	2

The items are scored 0 (not reported), 1 (reported but inadequate) or 2 (reported and adequate). The global ideal score being 16 for non-randomized studies and 24 for comparative studies

**Table 8:** Defect location

Articles	Defect / tumor location	FF	PF	*p*-value
Goyal, 2017	Cutaneous/lateral skull base	60 (10.2%)	56 (26.9%)	**< 0.001**
	Oral cavity	154 (26.2%)	32 (15.4%)	
	Oropharynx	42 (7.1%)	4 (1.9%)	
	Laryngectomy/pharyngectomy	109 (18.5%)	58 (27.9%)	
	Mandibular	96 (16.3%)	9 (4.3%)	
	Sinonasal	49 (8.3%)	9 (4.3%)	
	Composite/multiple sites	79 (13.4%)	40 (19.2%)	
Kozin, 2016	Cutaneous defect	6 (21.4%)	15 (33.3%)	0.367
	Parotid/temporal bone	9 (32.1%)	16 (35.6%)	
	Total auriculectomy	4 (44%)	8 (50%)	0.071
Howard, 2016			4 (67%)	
	Lateral TBR	7 (78%)	14 (88%)	0.740
			5 (83%)	
	Subtotal TBR	2 (22%)	2 (13%)	0.309
			1 (17%)	
	Facial nerve sacrifice	6 (67%)	6 (38%)	**0.016**
			3 (50%)	
Li, 2016^a^	Tongue	18(75.00%)	12(70.59%)	ND
Zhang S, 2015	Tongue	7 (46.7%)^b^	**7 (58.3%)** ^**b**^	0.70
	Tongue and FOM	8 (53.3%)^b^	5 (41.7) ^b^	
Zhang X, 2014^a^	Tongue	43 (65.2%)	23 (34.8%)	0.331
	FOM	21 (80.8%)	5 (19.2%)	
	Gum	9 (90%)	1 (10%)	
	Buccal	4 (66.7%)	2 (33.3%)	
	Palate	2 (100%)	0 (0)	
Jing, 2014	Total laryngectomy	22 (100%) ^b^	27 (100%) ^b^	ND
Deganello, 2013	Oral cavity	9 (56.25%) ^b^	11 (55%)^b^	ND
	Oropharynx	7 (43.75%)^b^	9 (45.0%)^b^	
Fang, 2013	Buccal defects	20	24	ND
		12		
Hsing, 2011^a^	Tongue	35 (28.9%)	86 (71.1%)	**< 0.001**
	FOM	7 (53.8%)	6 (46.2%)	
	Lip	17 (94.4%)	1 (5.6%)	
	Gum	15 (34.9%)	28 (65.1%)	
	Buccal	101 (36.9%)	173 (63.1%)	
	Palate	7 (58.1%)	5 (41.7%)	
	Retromolar trigone	4 (40%)	6 (60%)	
Paydarfar, 2011	Tongue	14 (42.4%)^b^	12 (44.4%)^b^	0.90
	Tongue and FOM	10 (30.3%) ^b^	9 (33.3%) ^b^	ND
	FOM	9 (27.3%)^b^	6 (22.2%)^b^	0.90
Chan, 2011	Hypopharynx	24 (100%) ^b^	92 (100%) ^b^	ND
		86 (100%) ^b^		
Mallet, 2009	Oral tongue (OT)	16 (64%)	20 (44%)	0.17
	Base of tongue	2 (8%)	11 (24%)	
de Bree, 2007^a^	Lateral tongue	8 (20%)	8 (20%)	ND
	FOM	10 (25%)	10 (25%)	
	Base of tongue	3 (8%)	3 (8%)	
Chien, 2005	Buccal mucosal	11 (100%)	16 (100%)	ND
Chepeha, 2004	Oral cavity	38 (54%)	52 (48%)	NS
	Oropharynx	16 (23%)	45 (42%)	
	Hypopharynx	10 (14%)	11 (10%)	
	Neck	2 (3%)	2 (2%)	
	Other	6 (10%)	10 (9%)	
Petruzzelli, 2002	Upper aerodigestive tract + tracheotomy	24 (100%)	15 (100%)	ND
Amarante, 2000	Orbital	–	6 (8.8%)^b^	ND
	Parotid	–	3 (4.4%) ^b^	
	Middle 1/3 skull base	11 (22.4%)^b^	–	
	Oropharynx	–	4 (5.9%)^b^	
	Pharyngo-laringeal	–	34 (50%)^b^	
	Pharynx cer. Esoph.	3 (6.1%)^b^	–	
	Mandibular	–	3 (4.4%)^b^	
	Cervical	–	1 (1.5%)^b^	
	Brachial plexus	1 (2.0%)^b^	–	
Tsue, 1997^a^	Tongue	1 (20%)	1 (80%)	ND
	FOM	1 (20%)	4 (80%)	
	Lateral and posterior oropharynx	1 (25%)	3 (75%)	
	Base of tongue	15 (76%)	4 (24%)	
	Retromolar trigone or Alveolus	10 (71%)	4 (28%)	
	Tonsil	3 (27%)	8 (73%)	

*TBR* Temporal bone resection, *FOM* Floor of the mouth

^a^ The article describes the tumor location instead of the defect location

^b^ Percentage calculated relying on the data presented. Percentage not provided by the article

Bold = Statistically significant, *p*-value ≤ 0.05

**Table 9:** Demographic data

	Article	FF	PF	*p*-value
Age, mean ± SD or mean (range)	Goyal, 2017	64.0 ± 12.0	66.5 ± 12.9	**0.017**
	Kozin, 2016	64 ± 10.1	67.2 ± 10	0.1876
	Forner, 2016	65	63	NS
	Jing, 2014	64.8 (50–84)	67.4 (44–89)	ND
	Granzow, 2013	58.75 (44–77)	55.61 (34–83)	0.39
	Deganello, 2013	58.2 ± 6.32	69.6 ± 6.8	**< 0.01**
	Fang, 2013	58.0 (25–78)	72.4 (55–80)	**< 0.001**
		57.2 (46–72)		**< 0.001**
	Paydarfar, 2011	54.7 (38–75)	58.1 (34–82)	0.24
	Hsing, 2011	54.1 ± 9.6	54.5 ± 12.5	0.839
	Demirtas, 2010	60 (38–73)	65.3 (49–82)	ND
			58.5 (17.3%)	ND
	Mallet, 2009	52.7 ± 9.3	56.8 ± 8.7	0.07
	Chien, 2005	53.5 (40–77)	50.1(38–66)	ND
	Chepeha, 2004	58	58	NS
	Petruzzelli, 2002	58.2 (9.8)	64 (12.8)	ND
	Amarante, 2000	15–81	37–71	ND
	Kroll, 1997	56 ± 13	62 ± 12	**0.0046**
Age (median)	Sinha, 2017	65.9 (57.7–74.2)	67.9 (60.3–76.8)	**0.037**
	Howard, 2016	68.3	76.9	0.172 ^a^
			73.3	
	Tsue, 1997	63 (62 ± 2)	64 ^a^ (64 ± 2)	ND
Age > 50 years	Li, 2016	9(27.50%)	3(17.65%)	0.304
	Gieger, 2016	82%	85.4%	0.661
	Zhang S, 2015	12 (80%)^a^	10 (83.33%)^a^	1.00
	Zhang X, 2014	19 (76%)	6 (24%)	0.597
Male n (%)	Sinha, 2017	63.0%	71,40%	0.0794
	Kozin, 2016	78.6%	68.9%	0.43
	Li, 2016	17(70.83%)	17(100%)	**0.043**
	Gieger, 2016	68.0%	76.4%	0.338
	Howard, 2016	7 (77.8)	15 (93.8)	0.240
		7 (77.8)	6 (100)	
	Forner, 2016	75%	43%	NS
	Zhang S, 2015	10 (66.7%)^a^	9 (75%)^a^	0.69
	Zhang X, 2014	66 (88%)	31 (32.0%)	**0.018**
	Jing, 2014	19 (86.4%)^a^	26 (96.3%)^a^	ND
	Granzow, 2013	11 (69%)	13 (72%)	1.0
	Deganello, 2013	12 (75)	16 (80)	0.88
	Fang, 2013	9 (45%)	11 (45.83%)	ND
		8 (66.67%)		ND
	Paydarfar, 2011	22 (66.7%)^a^	20 (90.1%)^a^	0.38
	Hsing, 2011	39 (40.2%)	58 (59.8%)	0.745
	Demirtas, 2010	7 (58.33)	8 (100%)	ND
			7 (100%)	ND
	Mallet, 2009	19 (76%)	38 (84.4%)	0.58
	Chien, 2005	11(100%)	14 (87.5%)	ND
	Chepeha, 2004	54 (76%)	84 (78%)	NS
	Amarante, 2000	36 (73.47%)	49 (72.06%)	ND
	Tsue, 1997	16 (67%)	8 (27.6%)	ND

^a^Percentage calculated relying on the data presented. Percentage not provided by the article

Bold = Statistically significant, *p*-value ≤ 0.05

**Table 10:** Preoperative risk factors

	Article	FF	PF	*p*-value
Smoking	Gieger, 2016	62.0%	61.8%	0.985
	Jing, 2014	4 (18.18%)^a^	1 (3.70%)^a^	ND
	Granzow, 2013	9 (56%)	13 (72%)	0.5
	Mallet, 2009	23 (92%)	43 (98%)	ND
	Funk, 2002	13 (61.9%)	18 (85.7%)	0.159
Prior head and neck surgery	Goyal, 2017	189 (32.1%)	122 (58.7%)	**< 0.001**
	Gieger, 2016^a^	14.0%	30.9%	**0.039**
	Chepeha, 2004	25 (35%)	31 (29%)	**ND**
	Tsue, 1997	5 (17%)	11 (46%)	0.05
Systemic diseases (CVD, HTA, DM)	Fang, 2013	4 (20%)	20 (83.33%)	**< 0.001**
		3 (25%)		**< 0.001**
COPD	Sinha, 2017	12.2%	15%	0.4538
	Mallet, 2009	6 (24%)	10 (22%)	0.96
DM	Sinha, 2017	17.7%	15.8%	0.6900
	Granzow, 2013	5 (31%)	0	**0.02**
	Hsing, 2011	2 (33.3%)	4 (66.7%)	0.986
HTA	Gieger, 2016	56.0%	58.2%	0.821
	Granzow, 2013	6 (38)	5 (28)	0.7
CAD	Sinha, 2017	15.6%	18.8%	0.4162
	Granzow, 2013	1 (6%)	1 (6%)	1.0
	Mallet, 2009	6 (24%)	16 (36%)	0.46
DLP	Gieger, 2016	34.0%	34.6%	0.953
CHF	Sinha, 2017	3.4%	4.5%	0.5939
aFIB	Sinha, 2017	7.0%	15.0%	**0.0083**
Alcoolism	Mallet, 2009	24 (96%)	41 (95%)	ND
Other cancer	Mallet, 2009	3 (12%)	10 (22%)	0.46
Kaplan-Feinstein grade (severe comorbidity)	Funk, 2002	1 (4.8%)	5 (23.8%)	0.196

*aFIB* atrial fibrillation, *CAD* Cardiac artery disease, *CHF* Chronic heart failure, *COPD* Chronic obstructive pulmonary disease, *CVD* Cardiovascular disease, *DM* Diabetes mellitus, *DLP* Dyslipidemia, *HTA* Hypertension

Bold = Statistically significant, *p*-value ≤ 0.05

**Table 11:** ASA class and risk

Article		FF	PF	*p*-value
ASA class I-II n, (%)	Goyal, 2017	245 (41.6%)	56 (26.9%)	**0.001**
	Granzow, 2013	3 (18.75%)^a^	3 (16.67%)^a^	0.5
	Demirtas, 2010	9 (75%)^a^	5 (62.5%)^a^	ND
			5 (71.4%)^a^	ND
	Mallet, 2009	18 (75%)	33 (73.33%)	1.00
	de Bree, 2007	32 (80%)	37 (92.5%)	**0.028**
	Funk, 2002	10 (47.62%)	12 (57.14%)	0.226
ASA class III-IV n, (%)	Goyal, 2017	343 (58.2%)	152 (73.1%)	**0.001**
	Granzow, 2013	13 (81.25%)^a^	15 (83.33%)^a^	0.5
	Demirtas, 2010	3 (25%)^a^	3 (37.5%)^a^	ND
			2 (28.57%)^a^	ND
	Mallet, 2009	6 (25%)	12 (27%)	1.00
	de Bree, 2007	8 (20%)	3 (8%)	**0.028**
	Funk, 2002	11 (52.38%)	9 (42.86%)	0.226
ASA risk factor (mean ± SD)	Sinha, 2017	2.6 ± 0.03	2.8 ± 0.05	**0.0007**
	Forner, 2016	2.3	2.4	**0.05**
	Demirtas, 2010	2.25 ± 0.4	2.37 ± 0.5	ND
			2.28 ± 0.5	ND
	Kroll, 1997	2.74 ± 0.61	2.80 ± 0.68	0.8011

*ASA risk factor* scored using the American Society of Anesthesiology Scale (ASA)

^a^Percentage calculated relying on the data presented. Percentage not provided by the article

Bold = Statistically significant, *p*-value ≤ 0.05

**Table 12:** Staging and treatment data

	Article	FF	PF	*p*-value
Prior radiation	Goyal, 2017	272 (46.2%)	130 (62.5%)	**< 0.001**
	Kozin, 2016	12 (46.2%)	22 (48.9%)	0.824
	Howard, 2016	6 (67%)	7 (44%)	0.071
			3 (50%)	
	Gieger, 2016	98.0%	94.6%	0.356
	Jing, 2014	15 (68.2%)^a^	23 (85.2%)^a^	ND
	Granzow, 2013	4 (25%)	7 (39%)	0.5
	Paydarfar, 2011	4 (12%)^a^	0	0.14
	Mallet, 2009	3 (12%)	15 (33%)	0.09
	Chepeha, 2004	37 (52%)	56 (52%)	NS
	Tsue, 1997	8 (28%)	13 (54%)	**0.05**
	Kroll, 1997	49 (33.8%)	9 (28.1%)	0.5360
Prior chemotherapy	Kozin, 2016	7 (26.9%)	11 (25%)	0.859
	Jing, 2014	7 (31.8%)^a^	4 (14.8%)^a^	ND
	Granzow, 2013	1 (6%)	6 (33%)	0.09
	Paydarfar, 2011	1 (3%)^a^	1 (3.7%)^a^	0.14
	Tsue, 1997	0	1 (4%)	ND
T1	Zhang X, 2014	1 (50%)	1 (50%)	0.369
	Jing, 2014	7 (31.3%)^a^	8 (29.6%)^a^	ND
	Deganello, 2013	0	4 (20%)^a^	**< 0.01**
	Fang, 2013	0	3 (15%)^a^	ND
		0		ND
	Paydarfar, 2011	1 (3.0%)^a^	2 (7.4%)^a^	0.38
	Hsing, 2011	46 (42.2%)	63 (57.8%)	0.904
	Mallet, 2009	32% (8/25)	44% (20/45)	0.44
	Chien, 2005	0	9 (56.25%)^a^	NS
	Funk, 2002	1 (4.8%)	1 (4.8%)	NS
	Tsue, 1997	11 (37.93%)^a^	6 (25%)^a^	ND
T2	Zhang X, 2014	10 (76.9%)	3 (23.1%)	0.369
	Jing, 2014	6 (27.3%)^a^	10 (37.0%)^a^	ND
	Deganello, 2013	7 (43.8%)^a^	5 (25%)^a^	**< 0.01**
	Fang, 2013	7 (35%)^a^	12 (50%)^a^	ND
		4 (33.33%)^a^		
	Paydarfar, 2011	15 (45.45%)^a^	17 (62.96%)^a^	0.38
	Hsing, 2011	90 (35.3%)	165 (64.7%)	0.621
	Chien, 2005	6 (54.5%)^a^	7 (43.75%)^a^	NS
	Funk, 2002	1 (4.8%)	0	NS
T3	Zhang X, 2014	37 (66.1%)	19 (33.9%)	0.369
	Jing, 2014	7 (31.8%)^a^	2 (7.4%)^a^	ND
	Deganello, 2013	8 (50%)	8 (40%)	**< 0.01**
	Fang, 2013	3 (15%)	6 (25%)	ND
		2 (16.7%)		
	Paydarfar, 2011	11 (33.33%)^a^	6 (22.22%)^a^	0.38
	Hsing, 2011	38 (40.0%)	57 (60.0%)	0.621
	Chien, 2005	5 (45.45%)^a^	0	NS
	Funk, 2002	5 (23.8%)	5 (23.8%)	NS
T4	Zhang X, 2014	31 (79.5%)	8 (20.5%)	0.369
	Jing, 2014	1 (4.5%)^a^	7 (25.9%)^a^	ND
	Deganello, 2013	1 (6.25%)	3 (15%)	**< 0.01**
	Fang, 2013	9 (45%)	12.5%	ND
		6 (50%)		
	Paydarfar, 2011	6 (18.18%)^a^	2 (7.4%)^a^	0.38
	Hsing, 2011	12 (37.5%)	20 (62.5%)	0.621
	Chien, 2005	0	0	NS
	Funk, 2002	14 (66.6%)	15 (71.4%)	NS
T1-T2	Li, 2016	10 (41.67%)	8 (47.06%)	0.981
	Forner, 2016	5 (41.7%)^a^	6 (66.6%)^a^	ND
	Mallet, 2009	8 (32%)	20 (44%)	0.44
	Tsue, 1997	11 (37.93%)^a^	6 (25%)^a^	ND
T3-T4	Li, 2016	14(58.33%)	9(52.94%)	0.981
	Forner, 2016	7 (58.3%)^a^	3 (33.3%)^a^	ND
	Mallet, 2009	17 (68%)	25 (56%)	0.44
	Tsue, 1997	17 (58.6%)^a^	18 (75%)^a^	ND
Stage I-II	Chepeha, 2004	16 (23%)	23 (21%)	NS
Stage III-IV	Chepeha, 2004	85 (79%)	55 (77%)	NS
Surg +chemoradio	Li, 2016	8(33.33%)	7(41.18%)	0.854
	Fang, 2013	4 (20%)	5 (20.83%)	ND
		2 (16.67%)		ND
	Hsing, 2011	25 (39.7%)	38 (60.3%)	0.687
	de Bree, 2007	40 (100%)	37 (92.5%)	ND
	Chepeha, 2004	27 (38%)	46 (43%)	NS
	Paydarfar, 2011	16 (48.48%)^a^	12 (44.44%)^a^	**0.03**
Surg + radio	Zhang X, 2014	41 (51.9%)	17 (54.84%)	0.781
	Paydarfar, 2011	13 (39.39%)^a^	5 (18.5%)^a^	0.3
	Hsing, 2011	25 (39.7%)	38 (60.3%)	0.687
	Chepeha, 2004	27 (38%)	46 (43%)	NS
Tumor stage	Kroll, 1997	2.80 ± 0.94	3.14 ± 0.83	0.1106
Tumor reccurence	Kroll, 1997	44(32.6%)	11 (33.3%)	0.9350

Surg + chemoradio: Surgical resection and adjuvant chemoradiotherapy

Surg + radio: Surgical resection and adjuvant radiotherapy

^a^Percentage calculated relying on the data presented. Percentage not provided by the article

Bold = Statistically significant, *p*-value ≤ 0.05

**Table 13:** OR time, Hospital and ICU length and hospital cost

	Article	FF	PF	*p*-value
OR time, min (mean ± SD)	Sinha, 2017	421.4 ± 4.4	332.7 ± 10.7 min	**0.0001**
	Goyal, 2017	427.2 ± 92.3	310.8 ± 125.0	**0.001**
	Li, 2016	405 ± 107	365 ± 48	**< 0.05**
	Howard, 2016	683 (575–979)	544 (396–700)	**0.00817**
			601 (474–841)	0.2488
	Forner, 2016	552	347	**< 0.05**
	Zhang S, 2015	81 ± 8	55 ± 7	0.05
	Granzow, 2013	816.3 ± 148.9	587.9 ± 130.5	**0.0002**
	Deganello, 2013	570 ± 96	444 ± 54	0.14
	Paydarfar, 2011	780	506.4	**0.001**
	Demirtas, 2010	204 ± 52	111 ± 18	ND
			153 ± 18	ND
	de Bree, 2007	692	462	**< 0.005**
	Tsue, 1997	684 ± 16	666 ± 20	**0.003**
OR time, hour (mean ± SD)	Kozin, 2016	8.1	6.7	**0.002**
	Fang, 2013	8.2 ± 2.7	5.4 ± 1.0	NS
		8.4 ± 1.7		NS
	Mallet, 2009	7.01 ± 1.19)	4.19 ± 0.57	**< 0.001**
	Smeele, 2006	12.5 ± 1.9	9.9 ± 1.5	**< 0.0001**
	Kroll, 1997	10.49 ± 2.06	9.39 ± 2.59	**0.029**
	Kroll, 1992	8.7	8.3	NS
OR time of > 600 min	Zhang X, 2014	59 (74.68%)	3 (9.68%)	**0.001**
Hospit length, days (mean ± SD)	Kozin, 2016	11	8.8	0.12
	Howard, 2016	9.8 (7–22)	4.75 (2–14)	**0.00424**
			6.5 (6–7)	0.15886
	Forner, 2016	15.4	12.4	**> 0.05**
	Zhang S, 2015	17 ± 2.5	12 ± 1.7	**< 0.05**
	Granzow, 2013	18.50 (9–59)	16.4 (5–57)	0.4
	Deganello, 2013	23.2 ± 7.5	26.5 ± 9.9	0.63
	Paydarfar, 2011	14.0	10.6	**0.008**
	Demirtas, 2010	16.0 ± 8.7	15.2 ± 8.7	ND
			17.0 ± 4.7	ND
	O’Neil, 2010	34.298	29.649	0.184
	Mallet, 2009	18.1 ± 6.7	23.2 ± 14.1	0.10
	de Bree, 2007	24	28	**0.005**
	Smeele, 2006	22.9 ± 11.5	22.8 ± 15.7	NS
	Chepeha, 2004	12	14	**0.006**
	Petruzzelli, 2002	6.9 ± 1.9	7.5 ± 4.3	ND
	Tsue, 1997	13 ± 1.4	13 ± 1.1	NS
	Kroll, 1997	13.2 ± 5.4	19.8 ± 11.5	**0.003**
	Kroll, 1992	11.3	21.2	**0.003**
ICU length, days (mean ± SD)	Forner, 2016	4.7	0.14	ND
	Granzow, 2013	5.6 (4–9)	1.8 (0–5)	**0.0001**
	Smeele, 2006	0.1 ± 0.5	0.28 ± 0.81	NS
	Tsue, 1997	3 ± 0.8	2 ± 0.3	NS
Hospital cost $	Kozin, 2016	SCAIF 32% less expensive thant FTT		**0.0001**
	Forner, 2016	43,617.60	18,158.40	ND
	Deganello, 2013	22,924	19,872	**0.043**
	Demirtas, 2010^a^	2963 ± 1715 ^a^	2053 ± 687^a^	ND
			2924 ± 100 ^a^	ND
	de Bree, 2007	48,097	51,963	ND
	Smeele, 2006	23,600^b^	20,400 ^b^	ND
	Petruzzelli, 2002	22,821.04 ±5062.81 ^a^	17,648.20 ± 7817.86 ^a^	ND
	Tsue, 1997	50,026 ± 4340	38,246 ± 1440	**0.003**
	Kroll, 1997	28,460 ± 8435	40,992 ± 1958	**0.001**

^a^US$

^b^CAN$

Bold = Statistically significant, *p*-value ≤ 0.05

**Table 14:** Post-operative complications

	Article	FF	PF	*p*-value
Any complications	Gieger, 2016	68.0%	36.4%	**0.001**
	Zhang X, 2014	13 (16.46%)	14 (45.16%)	**0.002**
	Jing, 2014	8 (36.36%)	12 (44.44%)	ND
	Granzow, 2013	7 (44%)	7 (39%)	1.0
	Paydarfar, 2011	25 (75.75%)^a^	10 (37.04%)^a^	ND
	Amarante, 2000	31%	0	
	Kroll, 1992	4 (13%)	17 (44%)	**0.0145**
Infection	Gieger, 2016	16.0%	7.3%	0.170
	Granzow, 2013	0	1 (6.25%)	1.0
	Mallet, 2009	4 (16%)	6 (13%)	1.0
Recipient site infection	Goyal, 2017	84 (14.3%)	19 (9.1%)	0.076
	Kozin, 2016	2 (7.14%)^a^	2 (4.44%)^a^	0.106
	Paydarfar, 2011	2 (6.06%)^a^	2 (7.41%)^a^	ND
	O’Neil, 2010	4 (5.2%)	2 (5.4%)	0.962
	Mallet, 2009	16% (4/25)	13% (6/45)	1.00
	Chepeha, 2004	2 (3%)	18 (17%)	**< 0.004**
Donor site infection	Goyal, 2017	23 (3.9%)	5 (2.4%)	0.43
	Kozin, 2016	3 (10.71%)^a^	2 (4.44%)^a^	0.106
	O’Neil, 2010	1 (1.3%)	1 (2.7%)	0.593
Donor site morbidity	Chan Y, 2011	1(4.2%)	7(7.6%)	ND
		2(2.3%)		ND
Fistula	Goyal, 2017	18 (3.1%)	17 (8.2%)	**0.005**
	Geiger, 2016	22.0%	7.3%	**0.039**
	Jing, 2014	5 (22.72%)^a^	6 (22.22%)^a^	ND
	Paydarfar, 2011	5 (15.15%)^a^	0	ND
	Chan Y, 2011	3 (12.5%)	22 (23.9%)	ND
		4 (4.6%)		ND
	O’Neil, 2010	2/77 (2.6)	3/37 (8.1)	0.179
	Demirtas, 2010	0	1 (12.5%)^a^	ND
	Mallet, 2009	2 (8%)	4 (9%)	1.00
	Smeele, 2006	3 (0.09%)^a^	1 (0.03%)^a^	ND
	Chepeha, 2004	4 (5%)	5 (5%)	0.74
	Amarante, 2000	0	14 (20.59%)^a^	ND
Abscess	Gieger, 2016	4.0%	7.3%	0.477
Dehiscence recipient or donor site	Gieger, 2016	44.0%	23.6%	**0.029**
	Jing, 2014	1 (4.55%)^a^	0	ND
	O’Neil, 2010	2 (2.6%)	1 (2.7%)	0.974
	Smeele, 2006	4 (12.5%)^a^	1 (1.47%)^a^	ND
Dehiscence recipient site	Kozin, 2016	4 (14.29%)^a^	6 (13.33%)^a^	NS
	Granzow, 2013	0	1 (5.55%)^a^	1.0
	Paydarfar, 2011	5 (15.15%)^a^	0	ND
	Smeele, 2006	1 (3.13%)^a^	1 (3.13%)^a^	ND
	Chepeha, 2004	0	11 (10%)	**< 0.008**
Dehiscence donor site	Kozin, 2016	0	4 (8.89%)^a^	NS
	Zhang S, 2015	1 (6.67%)^a^	0	ND
	Granzow, 2013	3 (18.75%)^a^	2 (11.11%)^a^	0.6
	Paydarfar, 2011	0	2 (9.09%)^a^	ND
	Smeele, 2006	3 (9.38%)^a^	0	ND
	Kozin, 2016	3 (10.71%)^a^	0	NS
	Jing, 2014	0	4 (14.81%)^a^	ND
	Granzow, 2013	0	0	1.0
	Demirtas, 2010	1 (8.33%)^a^	0	ND
Hematoma	Chepeha, 2004	4 (6%)	4 (4%)	0.70
	O’Neil, 2010	1 (1.3%)	1 (2.7%)	0.593
Hematoma Donor site	Howard, 2016	1 (11.11%)^a^	1 (6.25%)^a^	ND
			0	ND
	Smeele, 2006	0	1 (3.13%)^a^	ND
Hematoma recipient site	Paydarfar, 2011	2 (6.06%)^a^	2 (9.09%)^a^	ND
	Smeele, 2006	1 (3.13%)^a^	1 (3.13%)^a^	ND
Partial flap necrosis	Zhang S, 2015	0	2 (16.67%)^a^	ND
	Paydarfar, 2011	0	3 (13.64%)^a^	ND
	Smeele, 2006	1 (3.125%)^a^	2 (6.25%)^a^	NS
	Chepeha, 2004	2 (2.82%)^a^	12 (11%)^a^	**< 0.006**
	Amarante, 2000	0	11 (16.2%)^a^	ND
	Kroll, 1992	0	4 (10%)	0.1979
Total flap necrosis	Smeele, 2006	2 (6.25%)^a^	1 (3.125%)^a^	
	Amarante, 2000	3 (6.12%)^a^	1 (1.5%)^a^	ND
Partial or total flap necrosis	Mallet, 2009	1 (4%)	14 (31%)	**0.02**
Osteonecrosis	Goyal, 2017	36 (6.1%)	17 (8.2%)	0.33
	Gieger, 2016	24.0%	3.6%	**0.007**
Deep Vein Thrombosis (Inferious member)	Sinha, 2017	1.6%	2.2%	0.2775
	O’Neil, 2010	3 (3.9%)	1 (2.7%)	0.746
Venous obstruction (At site)	Paydarfar, 2011	1 (3.03%)^a^	0	ND
Late anastomotic stricture	Chan Y, 2011	3 (12.5%)	25 (27.2%)	ND
		2 (2.3%)		ND
Blood transfusion	Deganello, 2013	3 (19%)	4 (20%)	0.96

^a^Percentage calculated relying on the data presented. Percentage not provided by the article

Bold = Statistically significant, *p*-value ≤ 0.05

**Table 15:** Post-operative outcomes

	Article	FF	PF	*p*-value
Operation revision	Howard, 2016	1.6 (1–3)	0.6 (0–1)	**< 0.00001**
			1.3 (1–2)	NS
	Gieger, 2016	34%	9.1%	**0.003**
	Granzow, 2013	4 (25%)	3 (17%)	0.7
	Paydarfar, 2011	2	1	ND
	O’Neil, 2010	13 (16.9%)	3 (8.1%)	0.207
	Demirtas, 2010	3	0	ND
	Chepeha, 2004	6 (8%)	13 (12%)	0.60
	Amarante, 2000	7	0	ND
Flap failure	Howard, 2016	0	0	NS
			0	NS
	Zhang S, 2015	1 (6.7%)	0	ND
	Jing, 2014	1	0	ND
	Granzow, 2013	1	0	0.5
	Paydarfar, 2011	1	0	ND
	Mallet, 2009	1 (4%)	14 (31%)	**0.02**
	de Bree, 2007	1	0	ND
	Smeele, 2006	2	1	ND
Mortality at 30 days	Jing, 2014	0	1	ND
	Chepeha, 2004	0	6 (6%)	0.08
Mortality at 1-year	Granzow, 2013	0	0	1.0
	Funk, 2002	2 (20%)	6 (28.6%)	ND
Mortality at 2-year	de Bree, 2007	13 (33%)	16 (40%)	0.602

Bold = Statistically significant, *p*-value ≤ 0.05

**Table 16:** Quality of Life data

		Article	FF	PF	*p*-value
UW-QOL global		Li, 2016	55.14 ± 9.24	54.36 ± 8.13	0.965
		Zhang X, 2014	70.5 ± 16.7	67.3 ± 12.9	0.860
		Hsing, 2011	66.0 ± 18.5	57.8 ± 18.2	0.090
UW-QOL: Pain		Li, 2016	71.63 ± 9.91	72.94 ± 11.13	0.751
		Zhang X, 2014	86.2 ± 10.8	89.9 ± 11.4	0.425
		Hsing, 2011	76.8 ± 23.0	68.1 ± 27.2	0.138
UW-QOL: Swallowing		Li, 2016	44.00 ± 16.27	43.78 ± 4.95	0.741
		Zhang X, 2014	49.4 ± 14.7	51.3 ± 21.7	0.840
		Hsing, 2011	49.3 ± 37.2	48.6 ± 32.7	0.962
UW-QOL: Chewing		Li, 2016	42.45 ± 6.15	43.43 ± 12.37	0.817
		Zhang X, 2014	52.6 ± 17.1	59.4 ± 12.9	0.498
		Hsing, 2011	34.5 ± 39.0	33.6 ± 36.7	0.973
UW-QOL: Speech		Li, 2016	51.27 ± 11.24	52.63 ± 12.43	0.461
		Zhang X, 2014	57.5 ± 20.1	76.1 ± 13.3	**0.017**
		Hsing, 2011	66.7 ± 27.2	44.7 ± 35.0	**0.002**
UW-QOL: Apparence		Li, 2016	57.47 ± 11.44	68.54 ± 13.24	**0.0001**
		Zhang X, 2014	76.4 ± 18.6	70.3 ± 17.1	0.308
		Hsing, 2011	67.3 ± 25.0	69.8 ± 25.5	0.535
UW-QOL: Activity		Li, 2016	64.23 ± 9.52	63.73 ± 8.41	0.641
		Zhang X, 2014	71.9 ± 11.5	74.8 ± 10.2	0.710
		Hsing, 2011	67.9 ± 24.2	66.8 ± 27.9	0.760
UW-QOL: Recreation		Li, 2016	66.59 ± 11.62	67.26 ± 9.23	0.445
		Zhang X, 2014	72.1 ± 10.2	78.9 ± 11.2	0.590
		Hsing, 2011	69.1 ± 32.6	62.5 ± 32.2	0.221
UW-QOL: shoulder		Li, 2016	61.52 ± 7.83	54.65 ± 11.24	**0.0001**
		Zhang X, 2014	87.1 ± 14.4	65.6 ± 20.0	**< 0.001**
		Hsing, 2011	81.4 ± 14.7	50.5 ± 29.8	**< 0.001**
UW-QOL: Taste		Li, 2016	50.91 ± 10.64	51.24 ± 11.23	0.673
		Zhang X, 2014	48.4 (18.3)	52.9 (19.6)	0.713
		Hsing, 2011	55.0 ± 43.2	45.9 ± 39.6	0.226
UW-QOL: Salive		Li, 2016	45.48 ± 16.92	44.17 ± 12.78	0.723
		Zhang X, 2014	70.9 ± 9.5	72.3 ± 23.1	0.813
		Hsing, 2011	71.7 ± 34.8	73.8 ± 28.1	0.964
UW-QOL: Mood		Li, 2016	69.94 ± 9.51	68.31 ± 14.72	0.474
		Zhang X, 2014	76.0 ± 14.7	71.6 ± 18.8	0.114
		Hsing, 2011	76.2 ± 24.7	60.8 ± 32.8	**0.022**
UW-QOL: Anxiety		Li, 2016	70.57 ± 15.11	72.55 ± 15.19	0.219
		Zhang X, 2014	78.5 ± 9.64	86.4 ± 17.5	0.775
		Hsing, 2011	75.9 ± 26.3	68.9 ± 33.9	0.423
UW-QOL: Composite score		Hsing, 2011	66.0 ± 18.5	57.8 ± 18.2	0.090
Speech	Excellent	Zhang S, 2015	12 (80.0%)^#^	11 (91.7%)^#^	0.62
	Good		3 (20%)	1 (8.3%)	
	Poor		0	0	
	Always understandable	O’Neil, 201	17 (53.1)	4 (22.2)	**0.014**
	Usually understandable		14 (43.8)	9 (50.0)	
	Difficult to understand		1 (3.1)	5 (27.8)	
Swallowing full/regular diet at follow-up (vs soft, liquid) n (%)		Zhang S, 2015^§^	13 (86.7%)^#^	10 (83.3%)^#^	1.00
		Paydarfar, 2011^%^	19	20	0.60
		Chan Y, 2011^*^	8 (38.2%)	24 (35.8%)	ND
			52 (61.9%)		ND
		O’Neil, 2010^**^	17 (59.4%)	6 (33.3%)	0.202
		Tsue, 1997^***^	8 (34%)	4 (17%)	**0.02**
Preoperative mouth-open width distance (mean) cm		Fang, 2013	1.5–6.2 (4.6)	1.2–6.2 (4.8)	ND
			0.9–6.0 (3.5)		ND
		Chien, 2005	6.3–3.5 (5.7)	6.1–2.5 (5.1)	0.384
Postoperative mouth-open width		Fang, 2013	1.4–5.8 (4.3)	1.1–4.7 (3.2)	ND
			0.8–5.8 (3.3)		ND
		Chien, 2005	5.9–3.2 (5.2)	5.6–1.6 (3.6)	0.384
Mouth-open width change (%)		Fang, 2013	4.0–9.1%	8.3–47.5%	**< 0.001**
			3.3–11.1%		**< 0.001**
		Chien, 2005	4.8–9.8%	5–45.5%	**< 0.001**
G-tube dependence, n (%)		Smeele, 2006	21.8%	34.3%	NS
		Chepeha, 2004	10 (16%)	40 (42%)	**0.001**
Feeding tube for > 21 days		Mallet, 2009	8 (36%)	17 (42%)	0.84
Feeding tube at discharge		Tsue, 1997	20 (69%)	20 (83%)	NS
Feeding tube at follow up^e^			11 (39%)	17 (85%)	**0.002**

§ Follow-up = 6 months

% at most recent follow-up

* Regular PO follow-up, median follow-up period was 82 months

** Follow-up period, time not mention

*** Median follow-up was 298 days

# Percentage calculated relying on the data presented. Percentage not provided by the article

Bold = Statistically significant, *p*-value ≤ 0.05
